# Glytube: A Conical Tube and Parafilm M-Based Method as a Simplified Device to Artificially Blood-Feed the Dengue Vector Mosquito, *Aedes aegypti*

**DOI:** 10.1371/journal.pone.0053816

**Published:** 2013-01-14

**Authors:** André Luis Costa-da-Silva, Flávia Rosa Navarrete, Felipe Scassi Salvador, Maria Karina-Costa, Rafaella Sayuri Ioshino, Diego Soares Azevedo, Desirée Rafaela Rocha, Camila Malta Romano, Margareth Lara Capurro

**Affiliations:** 1 Departamento de Parasitologia, Instituto de Ciências Biomédicas, Universidade de São Paulo, São Paulo, São Paulo, Brasil; 2 Laboratório de Virologia, Instituto de Medicina Tropical, Universidade de São Paulo, São Paulo, São Paulo, Brasil; Universidade Federal do Rio de Janeiro, Brazil

## Abstract

*Aedes aegypti*, the main vector of dengue virus, requires a blood meal to produce eggs. Although live animals are still the main blood source for laboratory colonies, many artificial feeders are available. These feeders are also the best method for experimental oral infection of *Ae. aegypti* with Dengue viruses. However, most of them are expensive or laborious to construct. Based on principle of Rutledge-type feeder, a conventional conical tube, glycerol and Parafilm-M were used to develop a simple *in-house* feeder device. The blood feeding efficiency of this apparatus was compared to a live blood source, mice, and no significant differences (*p* = 0.1189) were observed between artificial-fed (51.3% of engorgement) and mice-fed groups (40.6%). Thus, an easy to assemble and cost-effective artificial feeder, designated “Glytube” was developed in this report. This simple and efficient feeding device can be built with common laboratory materials for research on *Ae. aegypti*.

## Introduction

Blood feeding of *Aedes aegypti*, the major dengue viruses’ vector, is a fundamental part of routine protocols to maintain mosquito colonies in insectary facilities since a blood meal is required for egg production by females [1]. After bioethical certification by animal-care committees [2], [3], anesthetized or immobilized live animals are used frequently as a source of blood for mosquitoes [4]. However, use of live animals is not possible in many circumstances. For example, the lack of animal facilities or an animal-use regulatory or permitting structure can limit blood provision. Furthermore, the “3 Rs” principles (reduction, refinement and replacement) in the scientific use of live animals [5] also need to be considered when feeding anautogenous mosquito species. Artificial feeders can be applied to replace live animals as blood sources [4]. Dengue virus infection of *Ae. aegypti* for experimental proposes is usually done with a titered viremic blood meal and requires an artificial blood-feeder system [6], [7].

There are numerous reports showing successful development and use of different devices to artificially feed blood-sucking invertebrate vectors of human pathogens [8]–[10]. Artificial feeding apparati for *Ae. aegypti* also are represented extensively in the literature [11]–[17]. These approaches share common features. Blood is placed between a heating element (used to mimic vertebrate blood temperature) and a thin membrane, which females penetrate with their proboscis to access and imbibe the blood. One of the most popular devices is Rutledge-type feeder with Parafilm-M® simulating the skin [11]. Several based versions are available, but a few number of these devices is easy to assemble with common materials available in research laboratories and cost-effective [15].

We developed a simple artificial membrane-feeding method using a standard conical tube and Parafilm M as a simplified and convenient apparatus to facilitate the *Ae. aegypti* artificial blood-feeding.

## Materials and Methods

### Ethics Statement

All experiments with mice were carried out in accordance with the guidelines of the Ethical Principles for Experiment on Animals adopted by Sociedade Brasileira de Ciência de Animais de laboratório (SBCAL) and approved by the Institutional Ethics Review Committee (Comissão de Ética no Uso de Animais – CEUA)-Universidade de São Paulo, protocol #014.

Experiments involving the use of human blood were conducted according to the principles expressed in the Declaration of Helsinki. Concentrated human erythrocytes type A stored in ACD (acid citrate dextrose), cited as “blood” in the experiments, were donated by Instituto de Hematologia de São Francisco LTDA (HEMASF). This already-existing collection was (HEMASF-A) received as anonymous sample from blood donor. The protocol was approved by the Institutional Review Board in Human Research (Comissão de Ética em Pesquisa com Seres Humanos do Instituto de Ciências Biomédicas/USP) and Comissão Nacional de Ética em Pesquisa – CONEP, protocol #503.

### *Aedes aegypti* Rearing

*Ae. aegypti* (Higgs white-eye strain**)** larvae, pupae and adults were reared in a local facility at the Institute of Biomedical Sciences, University of São Paulo, Brazil. Mosquitoes were maintained at 27±1°C, 75–80% relative humidity and with a 12∶12 h light:dark cycle. Larvae were fed on finely powdered fish food (Tetramin®) and adult mosquitoes were maintained *ad libitum* on solution of 10% sucrose (w/v). Pre-mated five-day old adult females were blood-fed using anaesthetized mice when required.

### Glytube Materials and Assembling the Device

**Figure 1:**
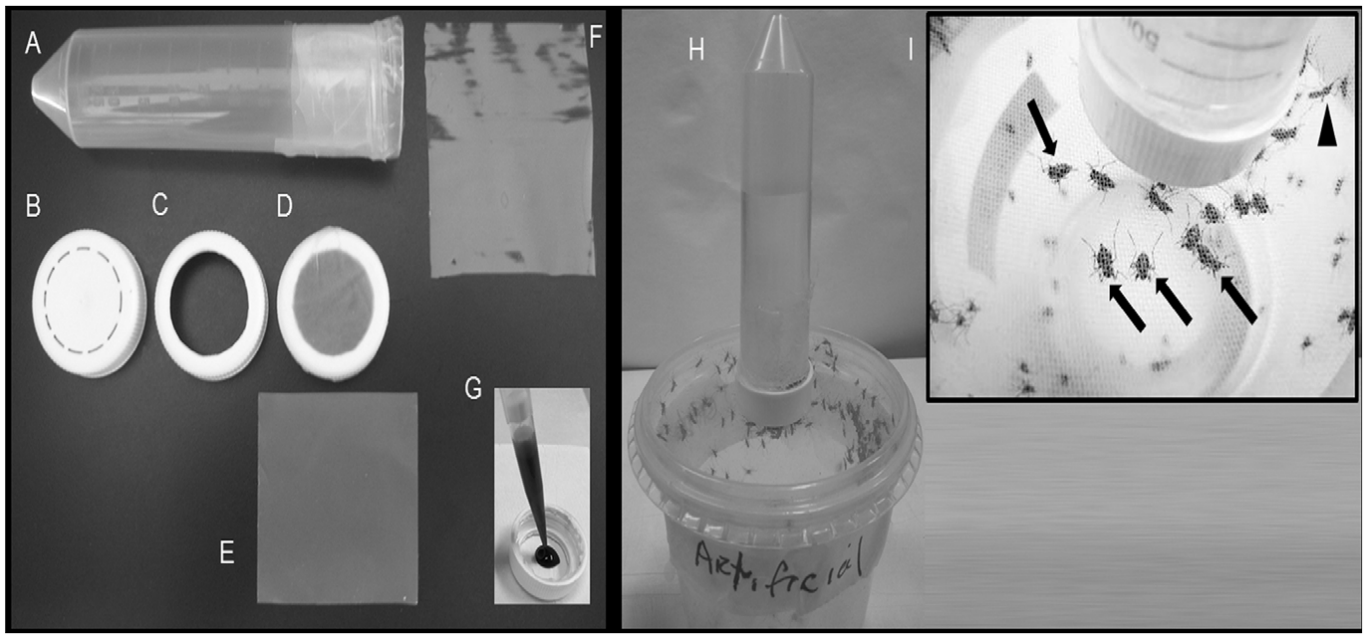
Materials used to assemble the Glytube blood feeder device. **A.** A conical tube (50 mL) filled with 40 mL warmed 100% glycerol and top sealed with Dura Seal^™^ heat-resistant sealing film. The sealing film is laterally held to the tube using a Parafilm-M^®^ thin strip (2.5 cm×5.0 cm). **B.** Screw cap of the conical tube. Dashed circular black line indicates the cap region where plastic is removed by cutting to generate the feeding element. **C.** Screw cap with 2.5 cm diameter hole. **D.** Screw cap covered externally with stretched Parafilm-M. A strip of Parafilm is fixing the feeding membrane to the cap. **E.** A piece of Parafilm-M (5 cm×5 cm) as a feeding membrane. Parafilm must be stretched to cover the screw cap. **F.** A piece of Dura Seal heat-resistant sealing film is used to sealing the conical tube filled with pre-heated 100% glycerol. **G.** Blood supplying the feeding element at internal side of the screw cap with the stretched Parafilm membrane. **H.** Heating and feeding elements assembled together to feed the *Ae. aegypti* females. **I.** Non blood-fed (black arrowhead) and artificially blood-fed females with dilated abdomens (black arrows).

**Figure 2:**
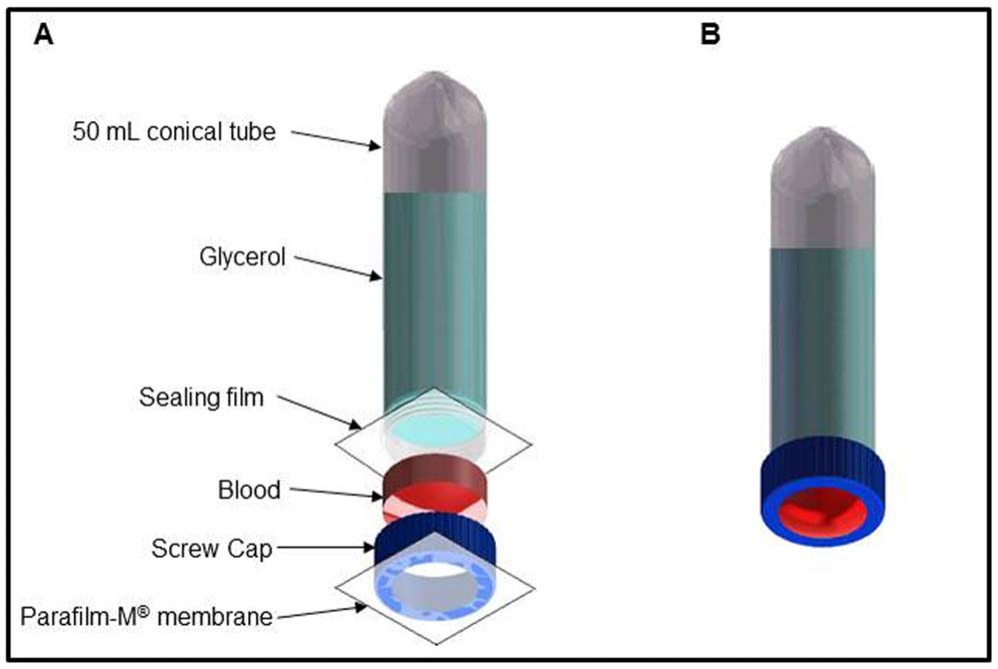
Schematic representation of the Glytube. **A.** Exploded drawing showing the materials used to prepare the device and elements order to assemble the *in-house* feeder. **B.** View of assembled Glytube before mosquito feeding.

The feeder, designated “Glytube”, is based on a 50 mL conical-bottomed tube with screw cap, Parafilm-M®, Dura Seal™ heat-resistant sealing film (or other laboratory sealing film) and 40 mL of 100% glycerol (Figures 1 and 2). An orifice 2.5 cm in diameter is cut in the center of the screw cap (Figures 1B and 1C) and a square of Parafilm membrane (5 cm×5 cm, Figure 1E) is rubbed on human skin to adhere natural odor ligands and improve its attractiveness to mosquitoes [18]. This membrane is stretched across the cap until becomes translucent and is placed over the screw cap (Figure 1D). The membrane edges are folded on the lateral gripping ribs at outer side of the screw cap. Another thin strip of Parafilm (2.5 cm×5.0 cm) is used to laterally fix the feeding membrane on the screw cap and the excess of stretched Parafilm is removed. This unit comprises the feeding element in which the internal part of screw cap is the blood reservoir (Figure 1D). The conical tube forms the heating element and is filled with 40 mL of 100% glycerol, the open end of the tube is sealed with Dura Seal film (10 cm×13 cm) and fixed laterally using a strip of Parafilm (2.5 cm×5.0 cm) (Figures 1A and 1F). The thermal conductivity of glycerol is low [19] and it works as a heated lid, keeping the blood temperature longer. The plastic that seals the tube (Figure 1F) provides the interface between blood and glycerol, facilitating heat transfer to the blood.

The feeding and heating elements are applied together to blood feed the mosquitoes (Figure 1H). Pre-heated blood (1.5 mL at 37°C) is placed in the internal side of the perforated screw cap with the fixed membrane (Figure 1G). Pre-heated glycerol (50°C) is transferred to the conical tube and the top is covered with Dura Seal film. The heating element is screwed to the cap containing the membrane and pre-heated blood. A complete screwing of the two elements is necessary to guarantee the contact between 1.5 mL blood volume and the heater element. The assembled apparatus is offered to the females confined to a plastic cup covered with fine-mesh marquisette. The Glytube is placed on the mesh-netted cup lids with feeding unit membrane in contact with the marquisette (Figure 1H).

### Blood and Conditions to Perform Mosquito Blood-feeding Experiments

All feeding experiments were done in an insectary maintained at 27±1°C. A total of 1.5 mL pre-heated (37°C) concentrated erythrocytes was delivered to the Glytube feeding element. For control groups, a plastic Petri dish with a 2.5 cm diameter hole (the same diameter of the orifice in the screw cap) in its center was used to provide the same accessible surface area for females to feed on mice. The anesthetized mice were placed on the Petri dish on top of the plastic cups containing the mosquitoes. A small region of the mouse’s abdomen (limited by the orifice) was exposed during feeding.

For artificial blood-feeding experiments, 100 female and 32 male (3∶1 ratio) pupae were divided in two groups and transferred into a plastic cup containing 100 mL distillated water and covered with fine-mesh marquisette. After adults’ emergence, distillated water was discarded and insects were kept on 10% sucrose solution. Four days after emergence, sucrose solution was removed and 5 days-old pre-mated females were allowed to blood-feed for 30 minutes on anaesthetized mice (Balb/c strain) (control group) or on Glytube device (experimental group). After 30 minutes, CO_2_ anaesthetized mosquitoes were maintained on ice and engorged females were counted. To obtain feeding efficiency, these experiments were done in biological triplicates (independent hatched postures), two with experimental triplicate groups and another one with experimental sixplicate groups.

### Statistical Analysis and Programs

Statistical analyses were performed using GraphPad Prism® (version 5.00) for Windows (GraphPad Software, San Diego, CA, USA). The engorged female percentages between Glytube and mice-fed groups were used to estimate the statistical significance by Mann-Whitney test.

Glytube schematic representation and exploded drawing were done using Solidworks program (version 2012).

## Results and Discussion

**Figure 3:**
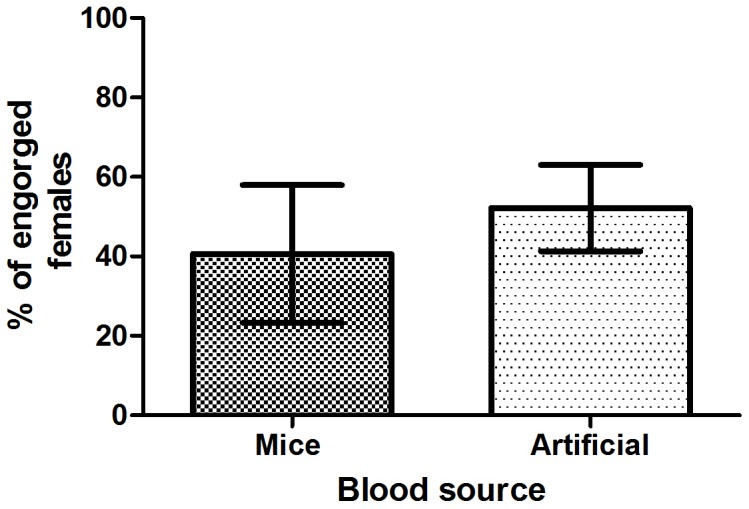
Blood-feeding efficiency of the Glytube feeder device compared with mice blood-fed females. The experiments were done in three biological replicates, two with three and another one with six independent experiments. To generate blood-feeding efficiency, the number of engorged females after 30 minutes of feeding was divided by total number of females allowed to feed. The numbers of females fed on Glytube or mice were not significantly different by Mann-Whitney test (p = 0.1189).

To evaluate Glytube feeding efficiency as an artificial feeder, the total number of engorged females was compared with that derived from controls in which females were fed for the same time (30 minutes) on anesthetized mice. Table 1 summarizes all the performed experiments. The Glytube feeder was a blood meal efficient source (51.3% [279/544] of females showed engorgement, Figure 1I) when compared to anaesthetized mice (40.6% [242/596]) and the difference of mean percentages between the Glytube and mice-fed groups are not significant (p = 0.1189, Figure 3).

**Table 1:** *Aedes aegypti* blood-feeding efficiency after 30 minutes between two methods.

Biological	Experimental	Blood Feeding Efficiency	
Replicate	Replicate	(Engorged/Total females)	
		Mice	Glytube
Hatching 1	Exp. 1	10% (5/50)	43,1% (22/50)
	Exp. 2	18% (9/50)	62% (31/50)
	Exp. 3	18% (9/50)	42% (21/50)
			
Hatching 2	Exp. 1	54,9% (28/51)	49% 24/49)
	Exp. 2	52,9% (27/51)	32% (16/50)
	Exp. 3	46,9% (23/49)	48% (24/50)
			
Hatching 3	Exp. 1	64% (32/50)	52,5% (21/40)
	Exp. 2	48,9% (22/45)	56,5% (26/46)
	Exp. 3	40% (20/50)	57,6% (19/33)
	Exp. 4	58% (29/50)	73,5% (25/34)
	Exp. 5	40,4% (21/52	60,9% (28/46)
	Exp. 6	35,4% (17/48)	48,9% (22/45)

Our results support the conclusion that the Glytube device is a useful method to artificially blood-feed *Ae. aegypti* females for colony maintenance in the insectary or when infected blood meals are needed in virus-challenge experiments. Reinforcing this assumption, Dengue virus-infected *Ae. aegypti* was obtained by using Glytube loaded with blood and virus supernatant mixture (data not shown). Also in a simple test, Glytube containing the same blood preparation described in this work was offered to two other Culicidae mosquito’s species - *Culex quinquefasciatus* (Culicinae) and *Anopheles aquasalis (*Anopheline*)* and engorged females were observed (data not shown), although the comparison with mice was not performed.

In this report, comparison of the artificial feeder efficiency with anaesthetized mice was done to show that the Glytube can be used to replace live animals on *Ae. aegypti* blood-feeding. Comparing live blood source to artificial feeder is a prerequisite to validate a new system. Furthermore, other reports which published different artificial feeders also just compared feeding efficiency in relation to live animals [12], [17], [20].

Feeding efficiency is a relevant parameter which needs to be observed in development of artificial feeder devices. Unfortunately, the differences in experimental conditions between articles that showed other blood feeders are enormous and impose difficulties to establish feeding efficiency comparison between the available feeders with confidence. For example, time allowed to feed, age of females, live animal used as blood source in controls, blood source for experimental groups and blood preparation before loading the artificial device are critical.

Glytube feeding efficiency is not high as traditional glass feeders. However, glass feeders are defined as “more complex designs for membrane feeders” [4]. Moreover, increasing the number of Glytube devices to offer during the feeding time can improve the feeding efficiency showed by our results with one feeder. Accordingly, mice available surface to by bitten by females in the control groups was limited using a plastic dish with a 2.5 cm diameter hole (the same diameter of the orifice in the screw cap) to reproduce the same surface that Glytube offers to females (as explained in the Material and Methods section). Without this artifice, a higher level of feeding efficiency on mice is observed (data not shown).

Glytube is a simple system, easily constructed in any laboratory, and requires no special materials or complex heater elements. Since the defined pre-warmed temperatures for blood (37°C) and glycerol (50°C) showed comparable feeding response in relation to live animal during 30 minutes, this range is satisfactory to stimulate *Ae. aegypti* to feed [13]. Furthermore, Glytube is simple to wash and disinfect because the conical tube and cap are autoclavable materials, and can be reusable after sterilization. The feeding membrane and sealing film plastic are discarded as infectious waste and replaced. Finally, this system can be adapted for a range in volume of blood (using a 15 mL conical tube for small volumes) or more than one 50 mL conical tube can be used simultaneously to feed colonies with many mosquitoes, increasing the feeding membrane surface to be bitten by females, also reflecting in feeding efficiency.

Different types of blood feeders, made with a variety of materials, facilitate the accessibility of laboratories around the world to construct their devices. This diversity avoids the limitation to perform experiments involving the blood feeding process.

### Conclusions

Glytube is one of the simplest and low-cost artificial feeder systems available and one of the most accessible apparati to be assembled with common materials found in conventional laboratories.

## References

[1] Clements AN (1992) The biology of mosquitoes. London: Chapman & Hall. 509 pp.

[2] Gauthier C (2008) The institutional animal care committee: Keystone of international harmonization. Japanese Society for Alternatives to Animal Experiments: 157–161.

[3] Filipecki ATP, Machado CJS, Valle S, Teixeira MDO (2011) The Brazilian legal framework on the scientific use of animals. ILAR journal/National Research Council, Institute of Laboratory Animal Resources 52: E8–15. 10.1093/ilar.52.1.e8 21447857

[4] Benedict MQ (2009) Bloodfeeding: Membrane Apparatuses and Animals. Methods in Anopheles Research. MR4. p. 288.

[5] Ferdowsian HR, Beck N (2011) Ethical and scientific considerations regarding animal testing and research. PloS one 6: e24059 doi:10.1371/journal.pone.0024059. 21915280 10.1371/journal.pone.0024059 PMC3168484

[6] Das S, Garver L, Ramirez JR, Xi Z, Dimopoulos G (2007) Protocol for dengue infections in mosquitoes (*A. aegypti*) and infection phenotype determination. Journal of Visualized Experiments: JoVE: 220. doi:10.3791/220. 10.3791/220 PMC2557096 18979018

[7] Salazar MI, Richardson JH, Sánchez-Vargas I, Olson KE, Beaty BJ (2007) Dengue virus type 2: replication and tropisms in orally infected *Aedes aegypti* mosquitoes. BMC Microbiology 7: 9. 17263893 10.1186/1471-2180-7-9 PMC1797809

[8] Chiang RG, Chiang JA (2010) Feeding through artificial membranes reduces fecundity for females of the blood-feeding insect, *Rhodnius prolixus* . Archives of Insect Biochemistry and Physiology 74: 103–113. 20513058 10.1002/arch.20365

[9] Huebner E, Harrison R, Yeow K (1994) A new feeding technique for experimental and routine culturing of the insect *Rhodnius prolixus* . Canadian Journal of Zoology 72: 2244–2247.

[10] Waladde SM, Young AS, Morzaria SP (1996) Artificial feeding of ixodid ticks. Parasitology Today (Personal ed) 12: 272–278. 15275192 10.1016/0169-4758(96)10027-2

[11] Rutledge LC, Ward RA, Gould DJ (1964) Studies on the feeding response of mosquitoes to nutritive solutions in a new membrane feeder. Mosquito News 24: 407–419.

[12] Cosgrove JB, Wood RJ, Petrić D, Evans DT, Abbott RH (1994) A convenient mosquito membrane feeding system. Journal of the American Mosquito Control Association 10: 434–436. 7807091

[13] Cosgrove J, Wood R (1995) Probing and gorging responses of three mosquito species to a membrane feeding system at a range of temperatures. Journal of the American Mosquito Control Association 11: 339–342. 8551304

[14] Tseng M (2003) A simple parafilm M-based method for blood-feeding *Aedes aegypti* and *Aedes albopictus* (Diptera: Culicidae). Journal of Medical Entomology 40: 588–589. 14680132 10.1603/0022-2585-40.4.588

[15] Rampersad J, Ammons D (2007) Versatile blood bags for laboratory feeding of mosquitoes. Journal of the American Mosquito Control Association 23: 149–152. 17847846 10.2987/8756-971X(2007)23[149:VBBFLF]2.0.CO;2

[16] Pothikasikorn J, Boonplueang R, Suebsaeng C, Khaengraeng R, Chareonviriyaphap T (2010) Feeding response of *Aedes aegypti* and *Anopheles dirus* (Diptera: Culicidae) using out-of-date human blood in a membrane feeding apparatus. Journal of Vector Ecology: Journal of the Society for Vector Ecology 35: 149–155. 20618661 10.1111/j.1948-7134.2010.00041.x

[17] Deng L, Koou SY, Png a B, Ng LC, Lam-Phua SG (2012) A novel mosquito feeding system for routine blood-feeding of *Aedes aegypti* and *Aedes albopictus* . Tropical Biomedicine 29: 169–174. 22543617

[18] Ghaninia M, Larsson M, Hansson BS, Ignell R (2008) Natural odor ligands for olfactory receptor neurons of the female mosquito *Aedes aegypti*: use of gas chromatography-linked single sensillum recordings. The Journal of Experimental Biology 211: 3020–3027 doi:10.1242/jeb.016360. 18775939 10.1242/jeb.016360

[19] Singh R (2004) Determination of thermal conductivity of high porosity organic foams at varying temperatures and pressures using thermal probe method. Indian Journal of Engineering & Materials Sciences 11: 125–129.

[20] Benzon GL, Apperson CS (1987) An electrically heated membrane blood-feeding device for mosquito colony maintenance. Journal of the American Mosquito Control Association 3: 322–324. 2904955

